# The small regulatory RNA DsrA silences the locus of enterocyte effacement of enteropathogenic *Escherichia coli* in an RpoS-dependent manner

**DOI:** 10.17912/micropub.biology.001409

**Published:** 2025-06-14

**Authors:** Brian Critelli, Zoe Mrozek, Alexa Mihaita, Lianna Long, Abigail Robinson, Shantanu Bhatt

**Affiliations:** 1 Department of Biology, Saint Joseph's University, 5600 City Avenue, Philadelphia, PA, 19131, United States; 2 Children's Hospital of Philadelphia, 3615 Civic Center Blvd., Philadelphia, PA, 19104, United States; 3 Perelman School of Medicine, 421 Curie Boulevard, University of Pennsylvania, Philadelphia, PA, 19104, United States

## Abstract

Attaching and effacing (A/E) pathogens adhere to intestinal cells (attachment) and destroy their microvilli (effacement). The A/E pathophenotype is encoded by a cluster of genes that are organized into the pathogenicity island called locus of enterocyte effacement (LEE). While transcriptional regulation of the LEE has been extensively interrogated in A/E pathogens, posttranscriptional regulation remains poorly understood. The RNA-binding protein Hfq and Hfq-dependent regulatory RNAs (sRNAs) play important roles in regulating the LEE posttranscriptionally. In a recent screen, we identified the Hfq-dependent sRNA DsrA as a novel riboregulator of the LEE in the A/E pathogen enteropathogenic *Escherichia coli* . Our findings suggest that DsrA globally silences the LEE by negatively regulating transcription of the *LEE1* -encoded master regulator Ler. The repression of *LEE1* is mediated through the stationary phase sigma factor, RpoS. Interestingly, our results contrast with what has been previously reported on the role of DsrA in EHEC, where the sRNA activates transcription from the *LEE1* promoter in an RpoS-dependent manner. The contrasting regulatory role of DsrA in EPEC and EHEC underscores the need for experimental validation of sRNA networks within each lineage, rather than inferring their function based on their roles in related bacteria.

**Figure 1. DsrA negatively regulates the LEE in the enteropathogenic Escherichia coli (EPEC) strain E2348/69 in an RpoS-dependent manner.**
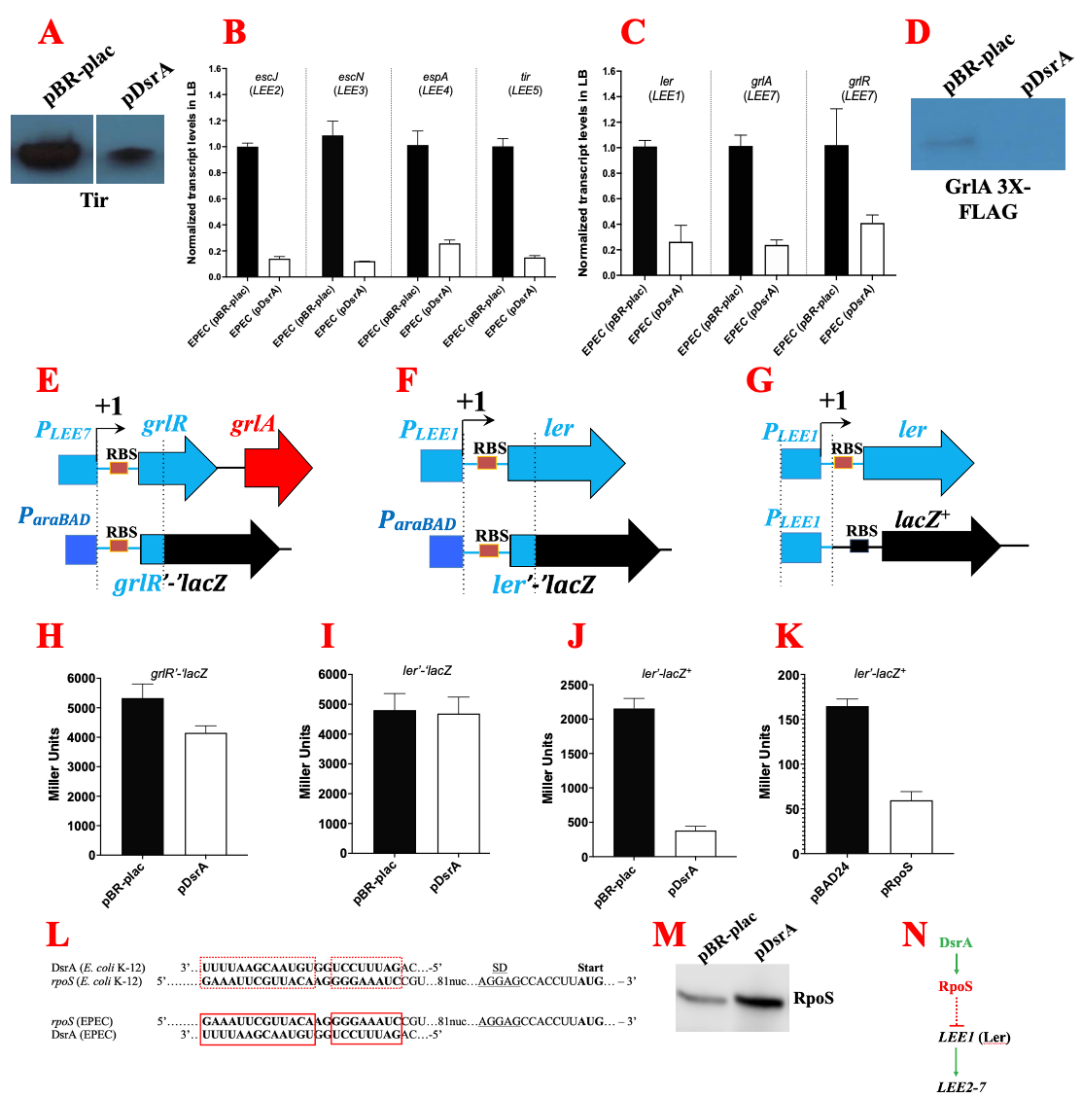
Overexpression of *dsrA* repressed the synthesis of the *LEE5* -encoded protein, Tir, as determined by Western blotting (A). The decrease in Tir levels resulted from a decrease in the *tir* mRNA levels, as determined by qRT-PCR (B). Besides *LEE5* , DsrA also repressed the steady-state mRNA levels specified from the *LEE2-4* operons (B). Gene expression from the *LEE2-5* operons is coordinately controlled by the Ler-GrlA-GrlR regulatory pathway, suggesting that one or more of these transcriptional regulators may be targeted by DsrA. Indeed, overexpression of *dsrA* repressed the synthesis of the *LEE1­-* encoded *ler* mRNA and the *LEE7-* encoded *grlRA* mRNA (C). The reduction in *grlA* resulted in the observed decrease in GrlA protein levels (D). DsrA did not affect β-galactosidase activity from a *grlR’-‘lacZ* (H) and *ler’-‘lacZ* (I) translational fusions, which harbor the 5’ UTR and the first 45 nucleotides of the *grlR* (E) and *ler* ORF (F), respectively, suggesting that DsrA does not base-pair to the 5’ leader regions of these mRNAs. Interestingly, DsrA inhibited β-galactosidase activity from a *ler’-lacZ* ^+^ transcriptional fusion (J) in which the *LEE1* promoter drives transcription of the full length *lacZ* gene (G), suggesting that DsrA indirectly controls transcription from the *LEE1* promoter by targeting a transcriptional factor. DsrA base-pairs to the 5’ UTR of the *rpoS* mRNA in the *E. coli* strain K-12 and exposes the ribosome binding site, which allows the ribosome to dock onto the mRNA and stimulate translation of RpoS (L). The RNA-coding region of DsrA as well as the 5’ UTR of *rpoS* , spanning the DsrA base-pairing region all the way to the start codon of *rpoS* , is conserved between the EPEC strain E2348/69 and the *E. coli* strain K-12 (L). This implies that, in EPEC too, DsrA is predicted to base-pair to the *rpoS* transcript (L). Consistent with the base-pairing, overexpression of *dsrA* positively regulated the production of RpoS in EPEC (M). Finally, overexpression of *rpoS* repressed transcription from the *LEE1* promoter, mimicking the repressive effect of *dsrA* (K). Model for DsrA-dependent regulation of the LEE (N).

## Description

Attaching and Effacing (A/E) pathogens infect individuals of all age groups and cause significant healthcare concerns globally (Mellies *et al.* , 2007, Bhatt *et al.* , 2016, Marshall *et al.* , 2020). Enteropathogenic *Escherichia coli* (EPEC) and enterohemorrhagic *E. coli* (EHEC) are the two prototypical members of the A/E morphotype. Other members of this group include *Escherichia albertii* and the mouse pathogen *Citrobacter rodentium* (Deng *et al.* , 2004, Bhatt *et al.* , 2019, Egan *et al.* , 2019) *.* A/E pathogens share a set of virulence factors, although there are differences between the diverse lineages as well. The most prominent virulence determinant that is conserved between them is the pathogenicity island locus of enterocyte effacement, that imparts to these pathogens their signature A/E pathophenotype (Deng *et al.* , 2001, Pallen *et al.* , 2005, Franzin & Sircili, 2015, Ooka *et al.* , 2015, Furniss & Clements, 2018, Bhatt *et al.* , 2019). The LEE is a ~35-40 kb genomic segment that is organized into 7 polycistronic operons ( *LEE1-7* ) and numerous monocistronic transcription units (Franzin & Sircili, 2015, Bhatt *et al.* , 2016). The LEE codes for a type 3 secretion system (T3SS), which upon assembly directly connects the bacterial cytoplasm to that of the intestinal cell, enabling the bacterium to inject effectors into the infected cell. The effector molecules are mechanistically and functionally diverse and primarily function to disrupt and manipulate host signal transduction pathways to ultimately aid bacterial colonization, survival, proliferation, and eventual dissemination (Croxen & Finlay, 2010, Deng *et al.* , 2010).

Transcriptional regulation of the LEE has been systematically characterized in A/E pathogens, and, for the most part, conserved transcriptional factors govern the LEE by similar mechanisms in the different members. For instance, the *LEE1* -encoded master transcriptional regulator Ler activates gene expression from the other transcription units of the LEE, including *LEE7* that specifies the transcriptional activator, GrlA, and its cognate anti-activator, GrlR (Deng *et al.* , 2004, Barba *et al.* , 2005, Padavannil *et al.* , 2013, Egan *et al.* , 2019). GrlA feeds back to further activate transcription from *LEE1* , whereas GrlR binds to GrlA and inhibits its activity (Jobichen *et al.* , 2007, Huang & Syu, 2008, Islam *et al.* , 2011, Padavannil *et al.* , 2013). This regulatory circuit is conserved in all the members of the A/E family. Conservation of transcriptional regulatory circuits also extends to shared non-LEE encoded transcriptional factors (Friedberg *et al.* , 1999, Sperandio *et al.* , 2002, Yona-Nadler *et al.* , 2003, Sircili *et al.* , 2004, Sharp & Sperandio, 2007). Beyond transcriptional regulation, LEE gene expression is further refined by posttranscriptional regulators such as the RNA chaperone protein Hfq that functions in concert with small regulatory RNAs (Hansen & Kaper, 2009, Shakhnovich *et al.* , 2009, Kendall *et al.* , 2011, Bhatt *et al.* , 2017, Egan *et al.* , 2019). The primary mechanism by which Hfq exerts its effect is as a molecular matchmaker, whereby Hfq simultaneously binds to an mRNA at one surface and an sRNA at another, bringing the two RNAs in proximity to sample each other (Updegrove *et al.* , 2016, Santiago-Frangos & Woodson, 2018). Complementary base-pairing between the sRNA and mRNA enables the former to regulate gene expression from the latter, which typically occurs by affecting mRNA stability and/or translation (Gottesman *et al.* , 2001, Gottesman & Storz, 2010). To date, dozens of Hfq-dependent sRNAs have been identified that either directly or indirectly regulate the LEE in A/E pathogens (Bhatt *et al.* , 2017, Bhatt *et al.* , 2017, Egan *et al.* , 2019, Melson & Kendall, 2019, Pearl Mizrahi *et al.* , 2021, Jia *et al.* , 2023, Muche *et al.* , 2023). Notably, regulatory circuits that are controlled by Hfq and Hfq-dependent sRNAs show varying degrees of evolution in different lineages of A/E pathogens. For instance, the three Hfq-dependent sRNAs, MgrR, RyhB, and McaS, that were originally identified as regulators of the LEE of EPEC, are predicted to regulate the LEE identically in *E. albertii* (Bhatt *et al.* , 2017, Egan *et al.* , 2019) *.* However, the Hfq-dependent sRNA Spot42 which regulates the LEE by affecting biosynthesis of the *ler* -inducer indole in EPEC is not predicted to regulate the LEE of *E. albertii* because the genes involved in indole biosynthesis have undergone genetic decay in the latter (Bhatt *et al.* , 2017, Egan *et al.* , 2019). Similarly, Hfq can function either as an activator or a repressor of the LEE depending on the genetic background. In the EPEC strain E2348/69 and in the EHEC strain EDL933, Hfq negatively regulates the LEE; however, in the EHEC strain 86-24 Hfq activates the LEE (Hansen & Kaper, 2009, Shakhnovich *et al.* , 2009, Kendall *et al.* , 2011, Bhatt *et al.* , 2017). Thus, regulatory circuits controlled by Hfq and Hfq-dependent sRNAs are evolutionarily flexible and must be experimentally verified in the different pathotypes.

In a recent screen, our lab isolated the Hfq-dependent sRNA, DsrA, as a repressor of the LEE-encoded protein Tir in EPEC ( Fig. 1A ). Specifically, controlled overproduction of DsrA from the plasmid pDsrA negatively regulated the synthesis of Tir when EPEC was cultivated in lysogeny broth – a medium that mimics environmental conditions that repress the LEE. Interestingly, deletion of *dsrA* did not significantly affect Tir synthesis under our experimental conditions. pDsrA, a derivative of the empty parental plasmid pBR-plac, expresses *dsrA* from the *P _llacO1_* promoter. The synthetic *P _llacO1_* promoter is tightly regulatable by the Lac repressor and inducible by IPTG over a >600-fold range (Lutz & Bujard, 1997, Guillier & Gottesman, 2006, Mandin & Gottesman, 2010). Throughout this study, the effects of overexpressing DsrA from pDsrA were quantified with reference to the control vector pBR-plac. Consistent with the reduced protein levels, the mRNA level of *tir* , which is encoded in the *LEE5* operon, was diminished upon overproduction of DsrA ( Fig. 1B ). Besides *LEE5* , many of the other *LEE* operons, particularly *LEE2-4* , also encode structural proteins and effector molecules of the T3SS (Franzin & Sircili, 2015, Platenkamp & Mellies, 2018). Thus, we assessed if gene expression from the other structural operons was dysregulated. This was done by assaying the abundance of a representative transcript encoded within each of these operons. DsrA globally silenced gene expression from each of these operons ( Fig. 1B ). Gene expression from the *LEE2-5* operons is coordinately regulated in a hierarchical manner by the Ler-GrlA-GrlR pathway (Mellies *et al.* , 2007, Bhatt *et al.* , 2016). Therefore, we assayed whether expression of one or more of these regulators was affected by DsrA. Overexpression of DsrA negatively regulated the synthesis of each of the three transcripts ( Fig. 1C ), suggesting that DsrA-dependent repression of *LEE2-5* occurs indirectly by controlling the synthesis of Ler, GrlR, and GrlA. Consistent with the observed reduction in *grlA* mRNA, the observed GrlA protein levels were also diminished in the DsrA overexpressor ( Fig. 1D ).

In EPEC, *grlR* and *grlA* are cotranscribed from the *LEE7* promoter to generate the bicistronic *grlRA* mRNA (Barba *et al.* , 2005), whereas *ler* is encoded by the first gene in the *LEE1* operon (Mellies *et al.* , 1999, Elliott *et al.* , 2000). Many Hfq-dependent sRNAs, including DsrA, exert their regulatory effects by base-pairing to target mRNAs, often within the 5’ untranslated leader region (UTR) of the first open reading frame (ORF) of the mRNA (Bhatt *et al.* , 2017). To test whether DsrA directly regulates the LEE by duplexing with either the 5’ UTR of *grlR* or *ler* , translational fusions to *lacZ* were engineered. Briefly, the entire 5’ UTR of *grlR* or *ler* along with the first 45 nucleotides of their respective ORF were fused in-frame to a truncated ‘ *lacZ* gene that lacks its 5’ UTR and some of the N-terminal codons, including the start codon ( Fig. 1E & 1F). The *grlR’-‘lacZ* and the *ler’-‘lacZ* chimeric genes were recombineered downstream of the *araBAD* promoter in the genetic background of the *E. coli* strain PM1205, which is a derivative of the K-12 MG1655 lineage ( Fig. 1E & 1F). This generates a reporter strain in which the *‘lacZ* gene is under the transcriptional control of the heterologous *araBAD* promoter and posttranscriptional control of the 5’ UTR of *grlR* or *ler.* Furthermore, the engineering of these fusions in *E. coli* K-12 instead of EPEC enabled us to uncouple the interregulatory control that Ler and GrlR/GrlA exert on each other. Overexpression of DsrA did not significantly affect β-galactosidase activity from either the GrlR’-‘LacZ ( Fig. 1H ) or Ler’-‘LacZ ( Fig. 1I ) translational fusions, suggesting that DsrA does not base-pair to the 5’ UTR of either *grlR* or *ler* . Next, we overexpressed DsrA in a reporter *E. coli* K-12 strain that harbors a transcriptional fusion in which the *LEE1* ( *ler* ) promoter drives transcription of the *lacZ* gene. Here, the *lacZ* gene retains its native 5’ UTR and the entire ORF ( Fig. 1G ). Interestingly, DsrA repressed β-galactosidase activity from the *ler’-lacZ ^+^* transcriptional fusion ( Fig. 1J ), suggesting that DsrA negatively regulates transcription from the *LEE1* promoter. Since DsrA base-pairs to mRNA targets to influence translation and/or mRNA stability, its effect on *LEE1* transcription must be indirect. This suggests that DsrA affects the expression of at least one transcriptional regulator of *LEE1* , which is conserved between the *E. coli* strain K-12 and the EPEC strain E2348/69. One such factor is the stationary phase sigma factor, RpoS.

In the *E. coli* strain K-12 substrain MG1655, DsrA activates the expression of RpoS. Specifically, DsrA base-pairs to the 5’ UTR of *rpoS* and exposes its ribosome binding site, enabling the ribosome to dock and initiate translation of RpoS while simultaneously protecting the *rpoS* mRNA from degradation ( Fig. 1L ) (Lease *et al.* , 1998, Majdalani *et al.* , 1998, McCullen *et al.* , 2010). However, whether DsrA exerts a similar effect on the *rpoS* homolog in the EPEC strain E2348/69 has not been tested. Pairwise alignment of DsrA from EPEC E2348/69 and *E. coli* MG1655 revealed that the sRNA is identical between the two. Furthermore, when the 5’ UTR of the *E. coli rpoS* mRNA was aligned with the homologous region of *rpoS* from EPEC, it was observed that the DsrA base-pairing region on *rpoS* was identically conserved in EPEC ( Fig. 1K ), suggesting that DsrA can similarly base-pair to the 5’ UTR of *rpoS* and stimulate its translation in EPEC. Consistent with this prediction, overproduction of DsrA led to an increase in the steady-state levels of RpoS in EPEC ( Fig. 1M ). Next, we tested whether the repressive effect of DsrA on the *LEE1* promoter was mediated via RpoS. To this end, the reporter strain harboring the chromosomal *ler’-lacZ ^+^* transcriptional fusion was transformed with the empty vector pBAD24 or its recombinant derivative pRpoS, that expresses *rpoS* from the arabinose inducible promoter *araBAD* . The plasmid pBAD24, like pBR-plac, provides tight regulation with minimal basal transcription, broad induction range, and short response time for a gene under the control of the *P _araBAD_* promoter (Guzman *et al.* , 1995). Regulated overexpression of *rpoS* repressed β-galactosidase activity from the *ler’-lacZ ^+^* transcriptional fusion ( Fig. 1K ), suggesting that RpoS negatively regulates transcriptional activity from the *LEE1* promoter. Notably, the basal level of β-galactosidase activity from the *ler’-lacZ ^+^* transcriptional fusion was significantly lower in the transformant harboring the empty vector pBAD24 compared to pBR-plac. Although, both pBAD24 and pBR-plac harbor the pBR322 origin of replication, the former lacks the *rop* gene, whose protein product limits the plasmid copy number (Guzman *et al.* , 1995, Cronan, 2006, Guillier & Gottesman, 2006). Thus, pBAD24 and pBR-plac exist in high and low copy numbers, respectively (Cronan, 2006). It is plausible that maintenance of pBAD24 requires considerable energy, which occurs at the expense of other cellular processes, thus limiting them. Alternatively, the presence of arabinose, to induce *rpoS* from pBAD24, may negatively regulate the *LEE1* promoter activity. In the EHEC strain ZAP193, arabinose and other 5-carbon sugars activate transcription from the *LEE* (Cottam et al., 2024) *.* It is plausible that in the EPEC strain E2348/69 arabinose has an antagonistic effect on the LEE. Controlled studies will shed more light on the precise role of arabinose in regulating the LEE of EPEC and how this compares to its observed role in EHEC.

In summary, our results suggest that the Hfq-dependent sRNA DsrA globally silences the LEE by indirectly repressing transcription of the LEE master regulator, Ler, via RpoS. Additionally, our findings also suggest that DsrA likely targets the *LEE1* promoter in an RpoS-independent manner because overexpression of DsrA had a more profound effect on transcriptional repression from *LEE1* promoter (β-galactosidase activity was reduced to ~17%) than overexpression of *rpoS* (β-galactosidase activity was reduced to ~35%). How does RpoS control transcription from *LEE1* ? It’s plausible that RpoS competes with other sigma factors that may be more efficient at recruitment and/or transcriptional initiation from the *LEE1* promoter. Alternatively, RpoS may indirectly repress the *LEE1* promoter by regulating transcription of a transitional regulator. Future studies in our lab are aimed at clarifying the precise mechanism by which DsrA and RpoS affect transcription from the *LEE1* promoter.

Our findings our notable in that they highlight the divergence of the regulatory role of DsrA in the two closely related A/E pathovars, EHEC and EPEC. A previous report showed that overproduction of *dsrA* activates transcription from the *LEE1* promoter of EHEC in an RpoS-dependent manner (Laaberki *et al.* , 2006). By contrast, our results suggest that DsrA exerts an antagonistic effect on the LEE in EPEC. Specifically, DsrA represses transcription from the *LEE1* promoter of EPEC in an RpoS-dependent manner. Reduced expression of *ler* , in turn, leads to reduced expression from the other *LEE* operons, which would be expected to reduce A/E lesion formation ( Fig. 1N ). In A/E pathogens, regulatory pathways that are discovered in one member are often assumed to function similarly in other members. Our results emphasize the importance of experimentally validating these generalizations, since regulatory circuits may undergo rewiring and influence bacterial physiology. This is especially relevant to regulatory networks that are controlled by small regulatory RNAs, since conserved regulators, such as DsrA, may not regulate conserved targets, such as the LEE, comparably in related A/E pathogens.

## Methods

**Bacterial strains, plasmids, primers & media:** Bacteria were streaked onto LB agar plates supplemented with ampicillin (100 µg/mL). Individual colonies were inoculated into LB broth supplemented with the same antibiotic and grown overnight at 37˚C/250 rpm for ~16-20 hours. Thereafter, the cultures were diluted 100-fold in the same medium, induced with the appropriate inducer, and grown to an OD600 of ~1.0. DsrA was induced by the addition of IPTG to a final concentration of 1 mM, whereas RpoS was induced by the addition of arabinose to a final concentration of 0.02%. Strains and plasmids used in this study are listed in Table 1 and oligonucleotides used are listed in Table 2.

**Chromosomal modifications:** The engineering of the chromosomal *P _araBAD_ -ler’-‘lacZ* , *P _araBAD_ -grlR’-‘lacZ* , and *grlA-* 3X-FLAG tagged translational fusions and the *P _LEE1_ -ler’-lacZ ^+^* transcriptional fusion has been described previously (Mellies *et al.* , 1999, Bhatt *et al.* , 2009, Bhatt *et al.* , 2017, Muche *et al.* , 2023). The reporter strains harboring the translational and transcriptional fusions were transformed with the parental empty vector, pBR-plac, or its recombinant derivative, pDsrA, that expresses *dsrA* . Additionally, the transcriptional reporter strain was also transformed with the empty vector pBAD24 or its derivative, pRpoS, which expresses the *rpoS* gene from *E. coli* under an arabinose inducible promoter.

**β-galactosidase assay:** β-galactosidase activity of the chromosomal *P _araBAD_ -grlR’-‘lacZ* and *P _araBAD_ - ler’-‘lacZ* translational fusions was assayed as described in our previous papers (Bhatt *et al.* , 2017, Muche et al., 2023), with slight changes. An individual bacterial colony was inoculated into 5 mL of LB supplemented with ampicillin (100 µg/mL) and grown overnight at 37˚C/250 rpm. The cultures were diluted 100-fold in 5 mL of the same medium that was additionally supplemented with arabinose (0.002-0.02%) and IPTG (1 mM). Arabinose induces transcription of the chimeric ‘ *lacZ* genes, whereas IPTG induces the transcription of *dsrA* from pDsrA. Cultures were grown at 37˚C/250 rpm to an optical density of ~1.0. β-galactosidase assays were performed on 100 µL of the permeabilized cell extracts. β-galactosidase activity of the chromosomal *P _LEE1_ -ler’-lacZ ^+^* transcriptional fusion was assayed using similar experimental conditions as those used for the translational fusions, with minor modifications. To assay β-galactosidase activity in the transformants harboring pBR-plac or pDsrA, the overnight grown cultures were diluted 100-fold in LB medium supplemented with ampicillin and IPTG (1 mM). For the transformants harboring pBAD24 or pRpoS, the overnight grown cultures were diluted 100-fold in LB medium supplemented with ampicillin and arabinose (0-002%-0.02%).

**RNA isolation and qRT-PCR:** Two individually isolated colonies of EPEC (pBR-plac) or EPEC (pDsrA) were each inoculated into separate test tubes containing 5 mL of LB supplemented with ampicillin and grown for ~16-20 hours at 37˚C/250 rpm. The following day the cultures were diluted 100-fold in 10 mL of LB supplemented with ampicillin and IPTG (1 mM) and grown to an optical density of ~1.0. RNA was isolated from 5 mL of the broth cultures by using the Trizol reagent, in accordance with the manufacturer’s (ThermoFisher) instructions. Contaminating DNA was removed from 1 µg of RNA by treating it with DNase I, as recommended (Ambion). The DNase treated RNA was concentrated by a second round of RNA purification, with the exception that the sample was not retreated with lysozyme. Quantitative reverse transcription real time PCR (qRT-RT PCR) was performed on 50 ng of RNA using the RotorGene SYBR Green RT-PCR kit (QIAGEN). The cycle threshold method 2 ^-∆∆Ct^ was used to compare the relative abundance of a transcript of interest in the *dsrA* overexpressor, EPEC (pDsrA), with respect to the empty vector containing parental strain, EPEC (pBR-plac). Briefly, the cycle threshold (C _t_ ) of the target transcript was normalized to the cycle threshold of the housekeeping transcript *rrsB* to generate the ∆C _t_ values for each sample. The ∆C _t_ for a specific transcript was averaged in the control strain EPEC (pBR-plac). Subsequently, the ∆∆C _t_ of each individual sample was calculated by subtracting the averaged ∆C _t_ of the specific transcript in the control strain from the ∆C _t_ values of the same transcript in the individual sample. The relative transcript abundance was then calculated using the formula 2 ^-ΔΔCt^ . This variation ensures that the mean relative expression of a target transcript is as close to 1 (100%) in the control samples. The primers used for qRT-PCR are listed in table 2. Each experiment was repeated on at least two separate occasions using 2 biological replicates. Similar results were obtained in each experiment.

**Preparation of cell lysates for Western Blotting:** The strains EPEC (pBR-plac) and EPEC (pDsrA) were probed for Tir and RpoS, whereas EPEC *grlA-* 3X-FLAG (pBR-plac) and EPEC *grlA* -3X-FLAG (pDsrA) were probed for 3X-FLAG tagged GrlA. Culture conditions for western blotting were identical to those described for RNA isolation and qRT-PCR above. The primary antibodies used were anti-Tir (5000-fold dilution; MyBioSource; Cat # MBS7049999), anti-RpoS (5000-fold dilution; Susan Gottesman, NIH), and anti-FLAG (5000-fold dilution; Sigma; Cat # A9469-1MG). The secondary antibody was conjugated to Horseradish peroxidase and used at 5000-fold dilution (Sigma; Cat #A0545-1ML). Each experiment was repeated on at least two separate occasions using 2 biological replicates. Similar results were obtained in each experiment.

**Table 1: Biological strains & Plasmids**

**Table:** 

**Strain**	**Description**	**Reference or Source**
EPEC	Wild type EPEC O127:H6 strain E2348/69	James Kaper
MG1655	Wild type *E. coli* K-12	Susan Gottesman
MC4100	*E. coli* K-12 MG1655 derivative	Lab stock
LS4922	EPEC (pBR-plac), Ap ^R^	(Bhatt *et al.* , 2017)
LS4931	EPEC (pDsrA), Ap ^R^	This study
LS1148	EPEC 2348/69 ɸ ( *grlA-* 3X-FLAG)	(Bhatt *et al.* , 2009)
LS5704	LS1148 (pBR-plac), Ap ^R^	This study
LS5724	LS1148 (pDsrA), Ap ^R^	This study
PM1205 = LS4767	* P _araBAD_ -cat-sacB-‘lacZ* mini-lambda, Cm ^R^ Tet ^R^ Suc ^S^	(Mandin & Gottesman, 2009)
LS4981	PM1205 *P _araBAD_ -grlR’-‘lacZ* , Cm ^S^ Tet ^S^ Suc ^R^	(Bhatt *et al.* , 2017)
LS5096	LS4981 (pBR-plac), Cm ^S^ Tet ^S^ Suc ^R^ Ap ^R^	(Bhatt *et al.* , 2017)
LS5063	LS4981 (pDsrA), Cm ^S^ Tet ^S^ Suc ^R^ Ap ^R^	This study
LS4978	PM1205 *P _araBAD_ -ler’-‘lacZ* , Cm ^S^ Tet ^S^ Suc ^R^	(Muche *et al.* , 2023)
LS5091	LS4978 (pBR-plac), Cm ^S^ Tet ^S^ Suc ^R^ Ap ^R^	(Muche *et al.* , 2023)
LS5089	LS4978 (pDsrA), Cm ^S^ Tet ^S^ Suc ^R^ Ap ^R^	This study
JLM164	MC4100 *ler’-lacZ ^+^*	(Mellies *et al.* , 1999)
LS5698	MC4100 *ler’-lacZ ^+^* (pBR-plac), Ap ^R^	(Muche *et al.* , 2023)
LS5734	MC4100 *ler’-lacZ ^+^* (pDsrA), Ap ^R^	This study
LS6021	MC4100 *ler’-lacZ ^+^* (pBAD24), Ap ^R^	This study
LS6024	MC4100 *ler’-lacZ ^+^* (pRpoS), Ap ^R^	This study
		
**Plasmids**		
pBR-plac	Parental vector for cloning, Ap ^R^	(Guillier & Gottesman, 2006)
pDsrA	pBR-plac derivative expressing *dsrA* under an IPTG inducible promoter, Ap ^R^	(Mandin & Gottesman, 2010)
pBAD24	Parental vector for cloning, Ap ^R^	(Guzman *et al.* , 1995)
pRpoS	pBAD24 derivative expressing *rpoS _E. coli_* under an arabinose inducible promoter	Nadim Majdalani

**Table 2: Oligonucleotides**

**Table:** 

**Name**	**Sequence**
SB2236 (5’ primer for *P _araBAD_ -grlR’-‘lacZ* )	ACCTGACGCTTTTTATCGCAACTCTCTACTGTTTCTCCATgcaatctggagaaaaagaaaggtct
SB2248 (3’ primer for *P _araBAD_ -grlR’-‘lacZ* )	TAACGCCAGGGTTTTCCCAGTCACGACGTTGTAAAACGACattgctaataaatataatgctatagatgcc
SB2234 (5’ primer for *P _araBAD_ -ler’-‘lacZ* )	ACCTGACGCTTTTTATCGCAACTCTCTACTGTTTCTCCATgaaaacagagaataataacattttaaggtgg
SB2247 (3’ primer for *P _araBAD_ -ler’-‘lacZ* )	TAACGCCAGGGTTTTCCCAGTCACGACGTTGTAAAACGACctgaatgtatggacttgttgtatgt
5’ *grlR* (downstream primer qRT-PCR)	TTAGCAATGAAGACTCCTGTGG
3’ *grlR* (downstream primer qRT-PCR)	AGAGAGAACCCCCTGATACAC
5’ *grlA* (upstream primer for qRT-PCR)	AGGCGGTTCCGATAGAAAGT
3’ *grlA* (downstream primer qRT-PCR)	GCCTCAAGATCATTTCGTTCC
5' *ler* (upstream primer for qRT-PCR)	GCAGTTCTACAGCAGGAAGCA
3' *ler* (downstream primer for qRT-PCR)	CGAGCGAGTCCATCATCAG
5' *tir* (upstream primer for qRT-PCR)	GCAGAAGACGCTTCTCTGAATA
3' *tir* (downstream primer for qRT-PCR)	CCCAACTTCAGCATATGGATTA
5' *espA* (upstream primer for qRT-PCR)	GCTGCAATTCTCATGTTTGC
3' *espA* (downstream primer for qRT-PCR)	GGGCAGTGGTTGACTCCTTA
5' *escJ* (upstream primer for qRT-PCR)	CCAAAGAAATGGACAAAAGTGG
3' *escJ* (downstream primer for qRT-PCR)	GCTGGGTGGGAAAATAACCT
5' *escN* (upstream primer for qRT-PCR)	CGGTATTGGGCAGCGTATC
3' *escN* (downstream primer for qRT-PCR)	GCCGATAAGAGCAAGGACAA
5’ *rrsB* (upstream primer for qRT-PCR)	CTTACGACCAGGGCTACACAC
3’ *rrsB* (upstream primer for qRT-PCR)	CGGACTACGACGCACTTTATG

## References

[R1] Barba J, Bustamante VH, Flores-Valdez MA, Deng W, Finlay BB, Puente JL (2005). A positive regulatory loop controls expression of the locus of enterocyte effacement-encoded regulators Ler and GrlA.. J Bacteriol.

[R2] Bhatt S, Edwards AN, Nguyen HT, Merlin D, Romeo T, Kalman D (2009). The RNA binding protein CsrA is a pleiotropic regulator of the locus of enterocyte effacement pathogenicity island of enteropathogenic Escherichia coli.. Infect Immun.

[R3] Bhatt S, Egan M, Critelli B, Kouse A, Kalman D, Upreti C (2018). The Evasive Enemy: Insights into the Virulence and Epidemiology of the Emerging Attaching and Effacing Pathogen Escherichia albertii.. Infect Immun.

[R4] Bhatt S, Egan M, Jenkins V, Muche S, El-Fenej J (2016). The Tip of the Iceberg: On the Roles of Regulatory Small RNAs in the Virulence of Enterohemorrhagic and Enteropathogenic Escherichia coli.. Front Cell Infect Microbiol.

[R5] Bhatt S, Egan M, Ramirez J, Xander C, Jenkins V, Muche S, El-Fenej J, Palmer J, Mason E, Storm E, Buerkert T (2016). Hfq and three Hfq-dependent small regulatory RNAs-MgrR, RyhB and McaS-coregulate the locus of enterocyte effacement in enteropathogenic Escherichia coli.. Pathog Dis.

[R6] Bhatt S, Jenkins V, Mason E, Muche S (2017). The Small Regulatory RNA Spot42 Inhibits Indole Biosynthesis to Negatively Regulate the Locus of Enterocyte Effacement of Enteropathogenic Escherichia coli.. Microorganisms.

[R7] Cottam C, White RT, Beck LC, Stewart CJ, Beatson SA, Lowe EC, Grinter R, Connolly JPR (2024). Metabolism of L-arabinose converges with virulence regulation to promote enteric pathogen fitness.. Nat Commun.

[R8] Cronan JE (2005). A family of arabinose-inducible Escherichia coli expression vectors having pBR322 copy control.. Plasmid.

[R9] Croxen MA, Finlay BB (2010). Molecular mechanisms of Escherichia coli pathogenicity.. Nat Rev Microbiol.

[R10] Deng W, de Hoog CL, Yu HB, Li Y, Croxen MA, Thomas NA, Puente JL, Foster LJ, Finlay BB (2009). A comprehensive proteomic analysis of the type III secretome of Citrobacter rodentium.. J Biol Chem.

[R11] Deng W, Li Y, Vallance BA, Finlay BB (2001). Locus of enterocyte effacement from Citrobacter rodentium: sequence analysis and evidence for horizontal transfer among attaching and effacing pathogens.. Infect Immun.

[R12] Deng W, Puente JL, Gruenheid S, Li Y, Vallance BA, Vázquez A, Barba J, Ibarra JA, O'Donnell P, Metalnikov P, Ashman K, Lee S, Goode D, Pawson T, Finlay BB (2004). Dissecting virulence: systematic and functional analyses of a pathogenicity island.. Proc Natl Acad Sci U S A.

[R13] Egan M, Critelli B, Cleary SP, Marino M, Upreti C, Kalman D, Bhatt S (2019). Transcriptional and posttranscriptional regulation of the locus of enterocyte effacement in Escherichia albertii.. Microb Pathog.

[R14] Elliott SJ, Sperandio V, Girón JA, Shin S, Mellies JL, Wainwright L, Hutcheson SW, McDaniel TK, Kaper JB (2000). The locus of enterocyte effacement (LEE)-encoded regulator controls expression of both LEE- and non-LEE-encoded virulence factors in enteropathogenic and enterohemorrhagic Escherichia coli.. Infect Immun.

[R15] Franzin FM, Sircili MP (2015). Locus of enterocyte effacement: a pathogenicity island involved in the virulence of enteropathogenic and enterohemorragic Escherichia coli subjected to a complex network of gene regulation.. Biomed Res Int.

[R16] Friedberg D, Umanski T, Fang Y, Rosenshine I (1999). Hierarchy in the expression of the locus of enterocyte effacement genes of enteropathogenic Escherichia coli.. Mol Microbiol.

[R17] Furniss RCD, Clements A (2017). Regulation of the Locus of Enterocyte Effacement in Attaching and Effacing Pathogens.. J Bacteriol.

[R18] Gottesman S, Storz G (2011). Bacterial small RNA regulators: versatile roles and rapidly evolving variations.. Cold Spring Harb Perspect Biol.

[R19] Gottesman S, Storz G, Rosenow C, Majdalani N, Repoila F, Wassarman KM (2001). Small RNA regulators of translation: mechanisms of action and approaches for identifying new small RNAs.. Cold Spring Harb Symp Quant Biol.

[R20] Guillier M, Gottesman S (2006). Remodelling of the Escherichia coli outer membrane by two small regulatory RNAs.. Mol Microbiol.

[R21] Guzman LM, Belin D, Carson MJ, Beckwith J (1995). Tight regulation, modulation, and high-level expression by vectors containing the arabinose PBAD promoter.. J Bacteriol.

[R22] Hansen AM, Kaper JB (2009). Hfq affects the expression of the LEE pathogenicity island in enterohaemorrhagic Escherichia coli.. Mol Microbiol.

[R23] Huang LH, Syu WJ (2008). GrlA of enterohemorrhagic Escherichia coli O157:H7 activates LEE1 by binding to the promoter region.. J Microbiol Immunol Infect.

[R24] Islam MS, Bingle LE, Pallen MJ, Busby SJ (2010). Organization of the LEE1 operon regulatory region of enterohaemorrhagic Escherichia coli O157:H7 and activation by GrlA.. Mol Microbiol.

[R25] Jia T, Wu P, Liu B, Liu M, Mu H, Liu D, Huang M, Li L, Wei Y, Wang L, Yang Q, Liu Y, Yang B, Huang D, Yang L, Liu B (2023). The phosphate-induced small RNA EsrL promotes E. coli virulence, biofilm formation, and intestinal colonization.. Sci Signal.

[R26] Jobichen C, Li M, Yerushalmi G, Tan YW, Mok YK, Rosenshine I, Leung KY, Sivaraman J (2007). Structure of GrlR and the implication of its EDED motif in mediating the regulation of type III secretion system in EHEC.. PLoS Pathog.

[R27] Kendall MM, Gruber CC, Rasko DA, Hughes DT, Sperandio V (2011). Hfq virulence regulation in enterohemorrhagic Escherichia coli O157:H7 strain 86-24.. J Bacteriol.

[R28] Laaberki MH, Janabi N, Oswald E, Repoila F (2006). Concert of regulators to switch on LEE expression in enterohemorrhagic Escherichia coli O157:H7: interplay between Ler, GrlA, HNS and RpoS.. Int J Med Microbiol.

[R29] Lease RA, Cusick ME, Belfort M (1998). Riboregulation in Escherichia coli: DsrA RNA acts by RNA:RNA interactions at multiple loci.. Proc Natl Acad Sci U S A.

[R30] Lutz R, Bujard H (1997). Independent and tight regulation of transcriptional units in Escherichia coli via the LacR/O, the TetR/O and AraC/I1-I2 regulatory elements.. Nucleic Acids Res.

[R31] Majdalani N, Cunning C, Sledjeski D, Elliott T, Gottesman S (1998). DsrA RNA regulates translation of RpoS message by an anti-antisense mechanism, independent of its action as an antisilencer of transcription.. Proc Natl Acad Sci U S A.

[R32] Mandin P, Gottesman S (2009). A genetic approach for finding small RNAs regulators of genes of interest identifies RybC as regulating the DpiA/DpiB two-component system.. Mol Microbiol.

[R33] Mandin P, Gottesman S (2010). Integrating anaerobic/aerobic sensing and the general stress response through the ArcZ small RNA.. EMBO J.

[R34] Marshall NC, Thejoe M, Klein T, Serapio-Palacios A, Santos AS, von Krosigk N, Kizhakkedathu JN, Stoynov N, Foster LJ, Overall CM, Finlay BB (2020). Master Sculptor at Work: Enteropathogenic Escherichia coli Infection Uniquely Modifies Mitochondrial Proteolysis during Its Control of Human Cell Death.. mSystems.

[R35] McCullen CA, Benhammou JN, Majdalani N, Gottesman S (2010). Mechanism of positive regulation by DsrA and RprA small noncoding RNAs: pairing increases translation and protects rpoS mRNA from degradation.. J Bacteriol.

[R36] Mellies JL, Barron AM, Carmona AM (2007). Enteropathogenic and enterohemorrhagic Escherichia coli virulence gene regulation.. Infect Immun.

[R37] Mellies JL, Elliott SJ, Sperandio V, Donnenberg MS, Kaper JB (1999). The Per regulon of enteropathogenic Escherichia coli : identification of a regulatory cascade and a novel transcriptional activator, the locus of enterocyte effacement (LEE)-encoded regulator (Ler).. Mol Microbiol.

[R38] Melson EM, Kendall MM (2019). The sRNA DicF integrates oxygen sensing to enhance enterohemorrhagic Escherichia coli virulence via distinctive RNA control mechanisms.. Proc Natl Acad Sci U S A.

[R39] Muche S, El-Fenej J, Mihaita A, Mrozek Z, Cleary S, Critelli B, Marino M, Yu W, Amos B, Hunter T, Riga M, Buerkert T, Bhatt S (2022). The two sRNAs OmrA and OmrB indirectly repress transcription from the LEE1 promoter of enteropathogenic Escherichia coli.. Folia Microbiol (Praha).

[R40] Ooka T, Ogura Y, Katsura K, Seto K, Kobayashi H, Kawano K, Tokuoka E, Furukawa M, Harada S, Yoshino S, Seto J, Ikeda T, Yamaguchi K, Murase K, Gotoh Y, Imuta N, Nishi J, Gomes TA, Beutin L, Hayashi T (2015). Defining the Genome Features of Escherichia albertii, an Emerging Enteropathogen Closely Related to Escherichia coli.. Genome Biol Evol.

[R41] Padavannil A, Jobichen C, Mills E, Velazquez-Campoy A, Li M, Leung KY, Mok YK, Rosenshine I, Sivaraman J (2013). Structure of GrlR-GrlA complex that prevents GrlA activation of virulence genes.. Nat Commun.

[R42] Pallen MJ, Beatson SA, Bailey CM (2005). Bioinformatics analysis of the locus for enterocyte effacement provides novel insights into type-III secretion.. BMC Microbiol.

[R43] Pearl Mizrahi S, Elbaz N, Argaman L, Altuvia Y, Katsowich N, Socol Y, Bar A, Rosenshine I, Margalit H (2021). The impact of Hfq-mediated sRNA-mRNA interactome on the virulence of enteropathogenic Escherichia coli.. Sci Adv.

[R44] Platenkamp A, Mellies JL (2018). Environment Controls LEE Regulation in Enteropathogenic Escherichia coli.. Front Microbiol.

[R45] Santiago-Frangos A, Woodson SA (2018). Hfq chaperone brings speed dating to bacterial sRNA.. Wiley Interdiscip Rev RNA.

[R46] Shakhnovich EA, Davis BM, Waldor MK (2009). Hfq negatively regulates type III secretion in EHEC and several other pathogens.. Mol Microbiol.

[R47] Sharp FC, Sperandio V (2007). QseA directly activates transcription of LEE1 in enterohemorrhagic Escherichia coli.. Infect Immun.

[R48] Sircili MP, Walters M, Trabulsi LR, Sperandio V (2004). Modulation of enteropathogenic Escherichia coli virulence by quorum sensing.. Infect Immun.

[R49] Sperandio V, Li CC, Kaper JB (2002). Quorum-sensing Escherichia coli regulator A: a regulator of the LysR family involved in the regulation of the locus of enterocyte effacement pathogenicity island in enterohemorrhagic E. coli.. Infect Immun.

[R50] Updegrove TB, Zhang A, Storz G (2016). Hfq: the flexible RNA matchmaker.. Curr Opin Microbiol.

[R51] Yona-Nadler C, Umanski T, Aizawa SI, Friedberg D, Rosenshine I (2003). Integration host factor (IHF) mediates repression of flagella in enteropathogenic and enterohaemorrhagic Escherichia coli.. Microbiology (Reading).

